# Survival and Energy Producing Strategies of Alkane Degraders Under Extreme Conditions and Their Biotechnological Potential

**DOI:** 10.3389/fmicb.2018.01081

**Published:** 2018-05-25

**Authors:** Chulwoo Park, Woojun Park

**Affiliations:** Laboratory of Molecular Environmental Microbiology, Department of Environmental Science and Ecological Engineering, Korea University, Seoul, South Korea

**Keywords:** extremophiles, alkane oxidizer, survival strategies, energy production, bioremediation

## Abstract

Many petroleum-polluted areas are considered as extreme environments because of co-occurrence of low and high temperatures, high salt, and acidic and anaerobic conditions. Alkanes, which are major constituents of crude oils, can be degraded under extreme conditions, both aerobically and anaerobically by bacteria and archaea of different phyla. Alkane degraders possess exclusive metabolic pathways and survival strategies, which involve the use of protein and RNA chaperones, compatible solutes, biosurfactants, and exopolysaccharide production for self-protection during harsh environmental conditions such as oxidative and osmotic stress, and ionic nutrient-shortage. Recent findings suggest that the thermophilic sulfate-reducing archaeon *Archaeoglobus fulgidus* uses a novel alkylsuccinate synthase for long-chain alkane degradation, and the thermophilic *Candidatus Syntrophoarchaeum butanivorans* anaerobically oxidizes butane via alkyl-coenzyme M formation. In addition, gene expression data suggest that extremophiles produce energy via the glyoxylate shunt and the Pta-AckA pathway when grown on a diverse range of alkanes under stress conditions. Alkane degraders possess biotechnological potential for bioremediation because of their unusual characteristics. This review will provide genomic and molecular insights on alkane degraders under extreme conditions.

## Background

Extremophiles are microorganisms that can not only survive but also prefer to grow under extremely harsh conditions (Rampelotto, 2013). Based on their habitat, extremophiles are classified as acidophiles (pH 0.5–5), alkaliphiles (pH 9–12), barophiles (approximately 0.1 MPa), psychrophiles (0°C–20°C), thermophiles (50°C–70°C), halophiles (5–20% salt). Extremophiles have evolved to possess special features for adapting to extreme conditions. For example, psychrophilic bacteria synthesize cold-shock proteins, which function as transcriptional enhancers and RNA-binding proteins, to protect RNA from numerous stressors (Wouters et al., 1999). In addition, many oligotrophs increase their surface area by synthesizing appendages for easy nutrient absorption and utilization of a wide range of substrates (Huyop and Cooper, 2012). Halophilic bacteria assimilate glycine betaine from the environment and produce osmoprotectants, such as trehalose or *N*-acetylglutaminylglutamine amide (Sagot et al., 2010).

**FIGURE 1:**
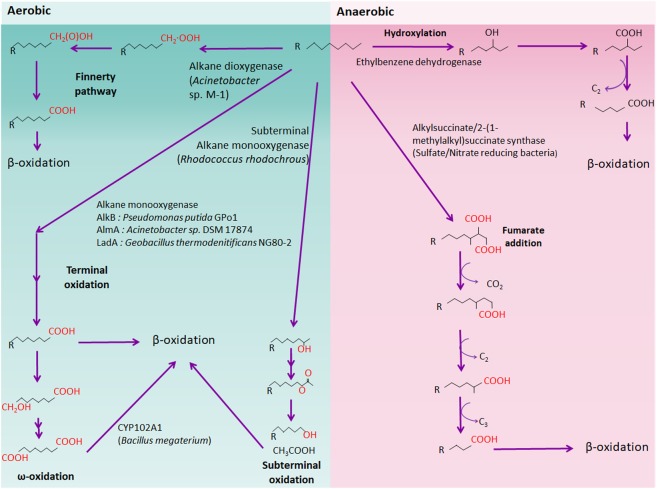
Schematic aerobic and anaerobic alkane degradation pathway. Aerobic (green background) and anaerobic (red background) alkane degradation pathways are shown. Each alkane oxidizer (e.g., alkane monooxygenase) is shown and representative bacteria are written in brackets.

*n*-alkanes, the major components of petroleum, can be metabolized aerobically or anaerobically by numerous microorganisms. Several alkane oxidation mechanisms have been documented till date, namely, terminal oxidation, subterminal oxidation, biterminal oxidation, and the Finnerty pathway. Alkane oxidation by aerobic bacteria produces fatty acids, which are subsequently metabolized via β-oxidation to generate acetyl-CoA (**Figure 1**). On the contrary, anaerobic alkane degradation is initiated by the addition of fumarate onto the terminal or subterminal carbon of alkane molecules, yielding alkylsuccinate derivatives as intermediates (**Figure 1**). Anaerobic alkane degradation was first proposed by So and Young, who demonstrated anaerobic alkane metabolism by fumarate- addition in the sulfate-reducing *Desulfatibacillum alkenivorans* AK-01 (So and Young, 1999; Rojo, 2009). Furthermore, recent studies also suggested that initial hydrocarbon activation by fumarate-addition occurs in syntrophic bacteria which produces acetate, CO_2,_ and/or H_2_ that are utilized by methanogens (Berdugo-Clavijo and Gieg, 2014). Anaerobic hydrocarbon-degrading syntrophic bacteria possess genes encoding an alkylsuccinate (*assA*) or a benzylsuccinate synthase (*bssA*) as well as genes for syntrophic processes, such as H_2_ and acetate production (Washer and Edwards, 2007; Kato S. et al., 2009; Walker et al., 2009; Fowler et al., 2012; Aitken et al., 2013; Cheng et al., 2013). However, several questions regarding the mechanism underlying anaerobic alkane degradation persist, such as the nature of all key enzymes and metabolic pathways for methanogenic alkane degradation by syntrophic bacteria and methanogen (Tan et al., 2015a).

Alkane monooxygenases have unique substrate ranges, which are classified into three categories based on the alkane chain length (van Beilen and Funhoff, 2007). Methane (C_1_) to butane (C_4_) conversion is catalyzed by methane monooxygenase (MMO)-like enzymes such as soluble MMO, particulate MMO, propane monooxygenase (PMO), and butane monooxygenase (BMO). In addition, integral membrane non-heme iron (AlkB) or cytochrome P450 enzymes (CYP153) oxidize pentane (C_5_) to hexadecane (C_16_), and alkanes longer than heptadecane are metabolized by a novel alkane dioxygenase, putative flavin-binding monooxygenase (AlmA), or long-chain alkane monooxygenase (LadA) (Maeng et al., 1996; Feng et al., 2007; Throne-Holst et al., 2007). However, a recent study showed that a novel Rieske-type alkane monooxygenase degrades pentane (C_5_) to tetracosane (C_24_) using NADH as a cofactor (Li et al., 2013). In addition, many bacteria possess multiple alkane hydroxylases (AH), and therefore, it is possible that a wide range of alkanes can be degraded by one microorganism (Whyte et al., 1998; Rozhkova-Novosad et al., 2007; Amouric et al., 2010; Liu et al., 2011; Park et al., 2017).

Biodegradation (transformation or mineralization) of a wide range of hydrocarbons, including aliphatic, aromatic, halogenated, and nitrated compounds, occurs in various extreme habitats. In addition, biodegradation of petroleum hydrocarbons by extremophiles has also been demonstrated under several conditions. For example, *Rhodococcus* sp. strain Q15, a psychrophile, showed short- and medium-alkane (C_10_ to C_21_) degradation at 5 °C. In addition, solid-phase microextraction–gas chromatography-mass spectrometry showed the co-appearance of 1-hexadecanol and 2-hexadecanol when strain Q15 utilized hexadecane, suggesting that Q15 assimilate alkanes via both the terminal and subterminal oxidation pathways (Whyte et al., 1998). A novel halophilic species, *Amycolicicoccus subflavus*, retains defensive genes against high salinity, osmotic stress, and poor nutrient availability, resulting in growth in the presence of 1–12% NaCl. Furthermore, genome analysis revealed that *Amycolicicoccus subflavus* possesses four AH (AlkB, CYP153, LadA, and PMO), which allows growth on C_10_ to C_36_ alkanes and propane (Nie et al., 2013). Thermophilic *Geobacillus* and *Aeribacillus* species, isolated from petroleum reservoirs and a hot spring, respectively, grew from 38°C to 68–70°Ñ, with maximum growth at 60°Ñ, and utilized C_10_–C_30_ alkanes as carbon source. Both strains are proven to have an *alkB*-type alkane monooxygenase. Two other *Geobacillus* strains (*G. toebii* B-1024 and 1017) also possess genes homologous to *lad*A and show activity similar to that of *G. toebii* B-1027 during long-chain alkane metabolism (Tourova et al., 2016).

Alkane-degrading extremophiles can be used in bioremediation of diverse oil-contaminated environments because of their special capabilities under extreme conditions. Cold-adapted hydrocarbon degraders have been applied to oil-polluted cold soil (Margesin et al., 2003; Aislabie et al., 2004; Wang J. et al., 2015) and wastewater (Margesin and Schinner, 1998; Gratia et al., 2009). In addition, psychrophiles from alpine habitats showed the highest degradation rate at 10°C within 8 days (40–60%) and at 4°C after 8 days (20–40%), showing high biodegradation efficiency (Margesin et al., 2000; Das and Chandran, 2011). Another well-studied example of bioremediation is the utilization of halophiles. Hydrocarbon biodegradation in the presence of high salinity is substantially valuable, not only from the economical aspect, but also in ecological and scientific studies. Recently, four *Pseudomonas aeruginosa* strains isolated from oil-contaminated saline soil were used to treat 10 g kg^-1^ of crude oil with more than 40% biodegradation efficiency in the presence of 300 mM NaCl after 120 days (Ebadi et al., 2017). *Marinobacter hydrocarbonoclasticus* showed high biodegradation activities of *n*-alkanes such as hexadecane (100%), eicosane (91%), and heneicosane (84%) in the presence of 4.6–20% NaCl (Gauthier et al., 1992). In another study, artificially weathered crude oil was proven to be degraded by microorganisms in a sandy salt marsh with a nitrogen–phosphorus–potassium (NPK) fertilizer (Lin et al., 1999). Several studies have demonstrated the feasibility of applying extremophiles to bioremediation and provided useful information for optimization of biodegradation efficiency.

However, despite the versatile potential of extremophiles in biodegradation, the molecular mechanisms and biological characteristics of these microorganisms have not been well-studied. This review discusses recent studies on alkane monooxygenase and 16S rRNA-based phylogenetic analysis of extremophiles, genotypic and phenotypic characteristics of pollutant-degrading extremophiles, and the ecological/advanced aspects of bioremediation. Overall, this review presents an updated overview of petroleum hydrocarbon degradation by microorganisms in different ecosystems.

## Phylogenetic Affiliation and Distribution of Alkane-Utilizing Extremophiles

### Extremophiles Possessing AlkB and CYP153 Family Alkane Hydroxylases

The genetic diversity of alkane hydroxylases has been extensively investigated under several conditions, including various oil-polluted and non-polluted environments. In particular, AlkB and members of the CYP153 family are widely distributed and have been extensively investigated in soil, marine environments, and groundwater (Sotsky et al., 1994; Knaebel and Crawford, 1995; Stapleton and Sayler, 1998; Luz et al., 2004; Kuhn et al., 2009).

#### Psychrophiles

Biodegradation of several petroleum components by indigenous cold-adapted microbial populations has been observed at low temperatures in hydrocarbon-contaminated Arctic and Antarctic soils. A large population of *Pseudomonas* species was observed in Arctic soil after diesel contamination. Furthermore, gene expression analysis confirmed that *Pseudomonas* and *Rhodococcus* species induce hydrocarbon degradation genes in Arctic biopile soils during bioremediation, indicating the importance of both species in oil-contaminated Arctic soil (Yergeau et al., 2012). DNA-based study showed that rhodococcal *alkB* genotypes (Rh *alkB*) are generally distributed in both contaminated and pristine Antarctic soils, whereas *alkB* from *Pseudomonas putida* (Pp *alkB*) was detected more in contaminated soils than in non-contaminated soils, implying that *Rhodococcus* spp. may be the predominant alkane-degradative bacteria in both polar soils, but *Pseudomonas* spp. may be enriched after contamination (Whyte et al., 2002). Populations of γ-proteobacteria such as *Colwellia*, *Marinomonas*, and *Glaciecola* appeared to be predominated in oil-contaminated Arctic fjord ice cores, (Brakstad et al., 2008). The 16S rRNA gene libraries of both non-contaminated and crude oil-contaminated seawater from sub-Antarctic areas showed the predominance of *Roseobacter*, *Sulfitobacter*, *Staleya*, *Glaciecola*, *Colwellia*, *Marinomonas*, *Cytophaga*, and *Cellulophaga*. However, non α- and γ- proteobacteria (𝜀-proteobacteria and Bacteroidetes) such as *Arcobacter*, *Polaribacter*, *Ulvibacter*, and *Tenacibaculum* started to appear when seawater was contaminated with hydrocarbons (Prabagaran et al., 2007). Therefore, α-, and γ-proteobacteria contribute maximally to alkane degradation in cold marine environments, whereas non α- and γ- proteobacteria have minor contribution.

#### Halophiles

The metagenomic database of a seawater sample from the Sargasso Sea (Venter et al., 2004) revealed the presence of *alkB* and *cyp153* (van Beilen and Funhoff, 2007), implying wide distribution of halophilic and halotolerant alkane degraders in the ocean environment. Other PCR-based study examining the Atlantic Ocean surface seawater revealed that both *alkB* and *cyp153* genes coexist in *Alcanivorax* and *Salinisphaera* species, whereas all *Parvibaculum* species possess only *cyp153* and discovered new culturable alkane-degraders belonging to *Brachybacterium*, *Idiomarina*, *Leifsonia*, *Martelella*, *Kordiimonas*, *Parvibaculum*, and *Tistrella* (Wang W. et al., 2010; Supplementary Table S1). Characterization of culturable *Alcanivorax* strains, *Marinobacter*, *Nocardioides*, *Parvibaculum* strains, originating from deep-sea hydrothermal vents, also revealed that only *Parvibaculum* strain possesses an alkane-oxidizing cytochrome P450 (CYP)-like protein to degrade alkanes (Bertrand et al., 2013). In subtropical seawater, *alkB* was detected in *Gallaecimonas*, *Castellaniella*, *Paracoccus*, and *Leucobacter* species, which shows a completely different bacterial community compared to the pelagic area (Wang L. et al., 2010), indicating that the distribution of alkane degraders varies with ocean conditions and geographical location. The number of α- and γ-proteobacteria (dominated by *Sphingomonadaceae*, *Rhodobacteraceae*, and *Chromatiales*) increased when crude oil was spilled in the supratidal and intertidal zones, on the other hand, γ- and δ-proteobacteria were more abundant in subtidal zones. The phylum Actinobacteria, particularly the genus *Rhodococcus*, was a key player in microbial response to the spillage especially in the supratidal sediment (Acosta-González et al., 2015). Likewise, no *alkB* sequence from Actinobacteria was detected in the marine metagenome despite a high proportion of *alkB* sequences from Actinobacteria in terrestrial and freshwater metagenomes (Nie et al., 2014).

#### Acidophiles

Although information on acidophilic alkane degraders is limited, some studies on alkane degradation at low pH have indicated the existence of hydrocarbon-degrading acidophiles. 16S rRNA library analysis of acidic soil from natural hydrocarbon seeps (pH 2.8–3.8) showed that majority of sequences were from heterotrophic acidophilic bacteria, including *Acidisphaera* and *Acidiphilium* species of α-proteobacteria, as well as the iron-and sulfur-oxidizing chemolithotroph *Acidithiobacillus* strain. A novel *Acidisphaera*-related strain C197 possesses *alkB* homologs (92.5% similarity with an *alkB* fragment from *Xanthobacter flavus*) and performs hexadecane metabolism (Hamamura et al., 2005). *Mycobacterium* is also a predominant bacterial genus found in extreme acidic sulfur block environments and alkane degradation of strain AG_S10_, an acidophilic *Mycobacterium*, was demonstrated. Interestingly strain AG_S10_ has both *alkB* and *cyp153* genes, implying the possibility of two alkane hydroxylase families in many acidophilic *Mycobacteria* strains (Ivanova et al., 2014).

### Extremophiles Possessing AlmA and LadA Family Alkane Hydroxylases

Flavin-binding family alkane hydroxylase (AlmA) and long-chain alkane monooxygenase (LadA) have recently been investigated; however, little is known about the distribution of either gene in the environment. Studies on functional diversity of alkane hydroxylase genes, including *almA* and *ladA* homologs, provided evidence that some psychrophiles possess AH for long-chain alkane degradation. Alignments with combined Pfam data sets showed the presence of *almA* homologs in *Paraglaciecola psychrophila* 170 (five copies) and *Psychrobacter cryohalolentis* K5 (one copy). On the contrary, a *ladA* candidate appears in the genomes of *Octadecabacter antarcticus* 307, *Octadecabacter arcticus* 238, and *Terriglobus saanensis* SP1PR4 (Bowman and Deming, 2014). These results hint at the occurrence of long-chain alkane metabolism at low temperatures, although the functionality of *almA* and *ladA* genes has not been shown experimentally. The diversity of *almA* in marine bacteria and oil-enrichment bacterial communities was investigated in three surface seawater samples from the South China Sea, Indian Ocean, and Atlantic Ocean (Wang and Shao, 2012). The acquired DNA sequences suggested two clades of *almA* gene. Class I *almA* contained a few sequences represented by the *Salinisphaera* and *Parvibaculum* genera, whereas class II was larger and more diverse and had many proteobacteria-related sequences, mainly from *Alcanivorax* and *Marinobacter*. However, metagenomic studies for detecting *ladA* in marine or saline conditions have not been conducted so far, although several marine hydrocarbonoclastic bacteria and halophilic hydrocarbon-utilizing bacteria harboring either AlmA- or LadA-type homologs, or both, have been reported (Liu et al., 2011; Nie et al., 2013; Meier et al., 2016). Thus, further investigation of long-chain alkane metabolism, especially the involvement of *almA* and *ladA*, under conditions of high salinity, are warranted.

Plasmid-born *ladA* gene was first investigated in *Geobacillus thermodenitrificans* NG80-2, indicating high possibility of horizontal gene transfer (HGT) of the *ladA* gene (Feng et al., 2007; Li et al., 2008). However, other member of *Geobacillus, G. thermoleovorans* B23 harbors three genes encoding LadA-type alkane hydroxylases (*ladA*α_B23_, *ladA*β_B23_, and ladB_B23_, collectively named the *ladAB* region) on its chromosome. Comparative genome analysis of relevant *Geobacillus* strains confirmed the absence of the *ladA* gene on the chromosome of *G. thermodenitrificans* NG80-2, but the presence of the *ladAB* gene island in many other *Geobacillus* strains (Boonmak et al., 2014).

**FIGURE 2:**
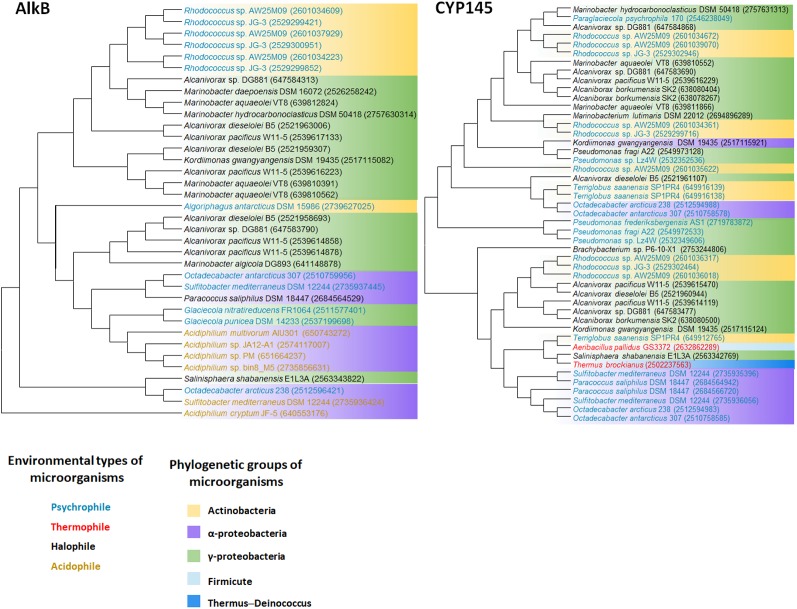
AlkB and Cytochrome P450 (CYP145) phylogenetic tree of extremophiles. Collected sequences were aligned and phylogenetic tree was drawn using the MEGA 6 program. Colored words indicate the following environmental types of microorganisms: blue, psychrophile; red, thermophile; black, halophile; yellow, acidophile. Colored backgrounds indicate the following phylogenetic groups: yellow, Actinobacteria; purple, Alphaproteobacteria; green, Gammaproteobacteria; light blue, Firmicute; dark blue, Thermus–Deinococcus.

**FIGURE 3:**
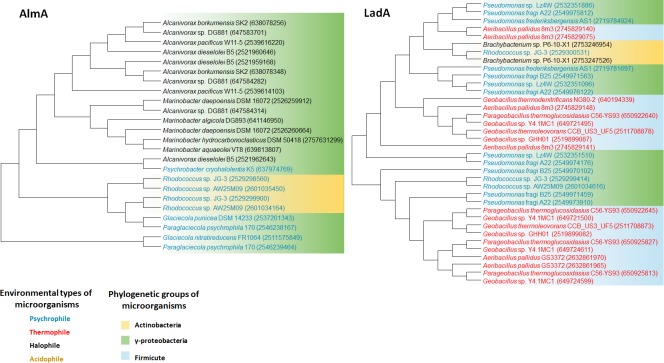
AlmA and LadA phylogenetic tree of extremophiles. Collected sequences were aligned and phylogenetic tree was drawn using the MEGA 6 program. Colored words indicate the following environmental types of microorganisms: blue, psychrophile; red, thermophile; black, halophile; yellow, acidophile. Colored backgrounds indicate the following phylogenetic groups: yellow, Actinobacteria; purple, Alphaproteobacteria; green, Gammaproteobacteria.

We retrieved whole genome data of extremophiles from the National Center for Bio-technology Information (NCBI)^1^ and Integrated Microbial Genomes (IMG)^2^ databases. Total 66 identified genomes were aligned and analyzed (Supplementary Table S1). *alkB* genes are widely distributed in aerobic alkane-degrading extremophiles (psychrophiles, halophiles, acidophiles, and thermophiles), although their association with HGT was not clear; nonetheless, the different evolution patterns of *cyp* and 16S rRNA, shown in **Figure 2**, indicate the occurrence of HGT. Interestingly, *alkB*, *cyp*, and *almA* were mostly detected in psychrophiles, whereas halophiles and thermophiles possess *ladA* (**Figure 3**). In addition, acidophilic alkane degraders possess only *alkB*, which is not present in the genome of thermophilic alkane degraders (**Figure 2** and Supplementary Table S1).

### Methanotrophic and Anaerobic Extremophiles

**FIGURE 4:**
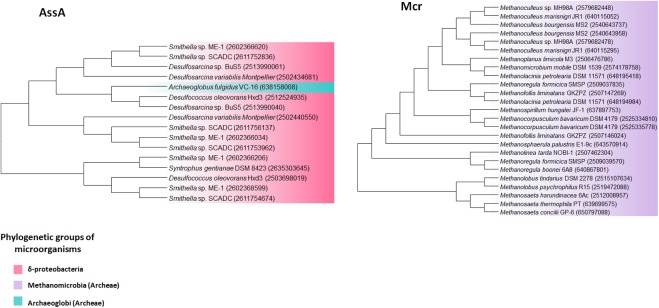
AssA and Mcr phylogenetic tree of extremophiles. Collected sequences were aligned and phylogenetic tree was drawn using the MEGA 6 program. Colored backgrounds indicate the following phylogenetic groups: pink, Deltaproteobacteria; purple, Metanomicrobia (archaea); green, Archaeoglobi (archaea).

Anaerobic methane oxidation (AOM) via reverse methanogenesis commonly occurs in anaerobic methane-oxidizing archaea (ANME) under anoxic environments, and was first identified in marine sediments where AOM was coupled to sulfate reduction. Therefore, ANME usually formed a community with sulfate-reducing bacteria (SRB) of δ-proteobacteria. ANME are generally classified into three categories: ANME-1 (sub-clusters a, and b) related to *Methanomicrobiales* and *Methanosarcinales*, ANME-2 (sub-clusters a, b, and c) related to cultivated members of *Methanosarcinales*, and ANME-3 related to *Methanococcoides* spp. (Timmers et al., 2017). However, all *mcr* genes (encoding methyl-coenzyme M reductase) in the class Methanomicrobia, such as *Methanoculleus, Methanoregula*, and *Methanosaeta* showed over 60% identity each other, which is indicative of its high conservation (**Figure 4**). Compared to samples containing only methane, samples from natural freshwater gas sources containing methane and sulfate, harbored ANME-2a/b such as *Methanolobus*, and AOM-associated archaea such as *Candidatus Methanoperedens nitroreducens*, as determined by archaeal 16S rDNA analysis (Timmers et al., 2016).

Methanogenic hydrocarbon-degrading δ-proteobacteria (*Syntrophus*/*Smithella*, *Desulfosarcina*/*Desulfococcus*), Firmicutes (*Desulfotomaculum*, *Desulfosporosinus*, and *Pelotomaculum*), and relatively few bacteria belonging to Spirochaetes, Bacteroidetes, Chloroflexi, and β-proteobacteria have been identified in hydrocarbon-contaminated aquifers (Tan et al., 2015a). In addition, previous reports showed that the γ-proteobacterial genus *Marinobacter*, and δ-proteobacterial genera *Syntrophus* and *Smithella*, are the predominant hydrocarbon degraders in samples from estuarine sand and oil sand tailings under methanogenic conditions (Gray et al., 2011; Siddique et al., 2012). Hydrocarbon degradation in marine environments is predominantly conducted by SRB, and on-site stable isotope probing revealed that *Desulfosarcina*/*Desulfococcus* (DSS) of δ-proteobacteria actively oxidize short- (butane) and long-chain (dodecane) alkanes. Although few SRB alkane degraders have been identified, several culture-dependent and independent studies at oil-contaminated seeps have indicated the presence of more diverse species than the currently known SRB (Kleindienst et al., 2014). In experimental condition, cultures enriched from oil sands tailings ponds included predominant δ-proteobacteria, such as *Desulfoglaeba*, *Desulfobacteraceae*, and *Geobacter* when short-chain alkane-degrading culture with a mixture of C_6_, C_7_, C_8_, and C_10_ *n*-alkanes as predominant organic carbon sources was transferred into sulfate-supplemented medium (Tan et al., 2015b).

## Survival Strategies of Extremophiles Under Harsh Condition

### Psychrophiles

Many DNA-binding proteins are present in extremophiles such as psychrophiles. For example, cold-shock proteins (Csp) play essential roles in DNA or RNA stabilization and membrane rigidity by regulating unsaturated fatty-acid synthesis (D’Amico et al., 2006). Interestingly, *Methanogenium frigidum*, a stenopsychrophilic archaea, possesses *csp* family of genes, which are generally not detected in archaeal (such as thermophilic and hyperthermophilic) genomes (Giaquinto et al., 2007). The Csp sequences of *M. frigidum* and *Escherichia coli*, are highly similar, and complementation of *csp*-knockout *E. coli* strain with *csp* gene from *M. frigidum*, results in growth recovery at low temperature, showing compatibility between archaeal and bacterial Csps. We observed that *Pseudomonas fragi* strains A22 and B25 (Supplementary Table S1) have the most abundant *csp* genes (6 genes) among putative alkane-degrading psychrophiles. However, six *csp* genes were also present in the genome of the mesophilic alkane degrader *Acinetobacter oleivorans* DR1. Thus, it is assumed that other strategies facilitate cold adaptation in psychrophiles regardless of the number of *csp* genes.

Most cold-adapted proteins in psychrophiles also show other characteristics, including high structure flexibility, low proportion of acyl chains in the cell membrane, and increased protein volume (Siddiqui and Cavicchioli, 2006). Chemical and biological reactions are slower at low temperatures, and therefore, the synthesis of cold-active enzymes with higher activity than mesophilic enzymes are required for maintaining an appropriate rate for essential metabolic reactions. To achieve high enzyme activity in cold conditions, the catalytic center must be flexible and unstable. Therefore, cold-adapted enzymes are heat labile and possess lower Gibbs free energy (*ΔG*) than mesophilic or thermophilic enzymes (Feller and Gerday, 2003; Siddiqui and Cavicchioli, 2006). Comparative sequence alignments of putative alkane hydroxylases from mesophiles and psychrophiles revealed that substitution by specific amino acids, such as alanine or glycine, contributes to increased flexibility of loops, bends, and α-helical structures of psychrophilic alkane hydroxylases. Furthermore, reports show that the flexible loops, bends, and helical regions of P450 from *Glaciecola psychrophila* are located in the center of three methionine residues, which is known to act as an alternative heme-binding site at low-temperatures (Bowman and Deming, 2014).

Trehalose production plays significant roles in resistance to freezing in cold environments. Observations from neutron diffraction, Raman spectroscopy, and inelastic neutron scattering revealed that trehalose disrupts the tetrahedral intermolecular network of water formed at low temperatures and thereby protects from ice damage. Image analysis based on field-emission transmission electron microscopy showed that trehalose was better than sucrose in preventing ice formation (Magazù et al., 2012). Currently, only psychrophilic *Rhodococcus* is known to produce trehalose lipid as a biosurfactant during alkane degradation (Rapp and Gabriel-Jürgens, 2003). However, there is a high possibility of trehalose lipid production by psychrophilic alkane degraders as trehalose synthesis can be induced by low temperature (Phadtare, 2004). Taken together, trehalose can be utilized as a cryoprotectant or biosurfactant by psychrophiles under cold conditions.

### Halophiles

Many halophilic microorganisms develop strategies for surviving in highly saline environments, such as transportation of inorganic ions (mainly potassium) for stabilizing external osmotic pressure or synthesis of metabolism-compatible osmolytes. Genome and expression analyses of the moderately halophilic alkane degrader, *Amycolicicoccus subflavus* DQS3-9A1^T^ showed upregulation of the KdpD/KdpE two component system (high-affinity K^+^ uptake system), Trk-type K^+^ transporter and Na^+^/K^+^ antiporter, MtrA/MtrB two component system (BetP regulator), and glycine/betaine transporter (BetP) in response to high salt stress (Nie et al., 2013). Trk-type K^+^ transporter, one of main K^+^ transporters, requires three genes for cellular K^+^ uptake, namely, *trkA* (1,374 bp, encoding cytoplasmic NAD^+^/NADH binding protein)*, trkH* (1,449 bp, encoding Trk transporter), and *trkI* (1,479 bp, encoding Trk transporter). Genome data show that several halophilic alkane degraders, such as *Paracoccus saliphilus*, *Marinobacterium lutimaris, Salinisphaera*, and *Marinobacter hydrocarbonoclasticus*, possess multiple Trk potassium uptake proteins, implying that Trk transporter provides osmolarity balance to halophiles during alkane degradation under high salinity conditions.

Several classes of organic compatible solutes, such as amino acids, alcohols, sugars, and derivatives act as organic osmoprotectants, which can be classified into three chemical categories: zwitterionic, non-polar, and anionic solutes (Roberts, 2005). These organic osmoprotectants are regulated by extracellular salt concentration. The synthesis rate of trehalose in *Haladaptatus paucihalophilus* decreases with increase in salinity; on the contrary, the intracellular concentration of glycine betaine increases due to import at high salinities, suggesting dynamic regulation of two compatible solutes for osmoadaptation (Youssef et al., 2014). The obligate hydrocarbon bacterium, *Alcanivorax borkumensis* SK2, synthesizes and accumulates ectoine to maintain osmolality under hyperosmosis, which promotes active metabolism and maintains cell integrity albeit low hydrocarbon degrading performance, thereby protecting cells from damage due to hyperosmosis (Scoma and Boon, 2016).

Undesirable interactions that destroy internal microbial proteins by dehydration can be avoided by controlling net charge. Compared to non-halophilic proteins, halophilic proteins contain several glutamate and aspartate residues on their surfaces, which provides high water solubility in the presence of salinity (Edbeib et al., 2016). Although comparative sequence alignment and structural studies of halophilic alkane hydroxylases have not been performed so far, we speculate that large numbers of glutamate and aspartate on their surfaces distributed to the stabilization of alkane hydroxylases.

### Acidophiles

Specific adaptation to and metabolism of alkane degradation have not been extensively studied in acidophiles. Acidophiles possess several systems for surviving in highly acidic conditions (Baker-Austin and Dopson, 2007), such as a cytoplasmic buffering, DNA protection and repair, blocking of extracellular proton uptake, and organic acid utilization (Babu et al., 2015). Buffer capacity in the cytoplasm is determined mainly by amino acids, such as glutamate and arginine, which sequester protons. An acid-resistant *E. coli* strain showed that decarboxylated amino acids which require intracellular proton consumption enhanced internal pH, resulting in maintenance of pH homeostasis. However, the buffering capacities of acidophilic bacteria *Bacillus acidocaldarius* and other neutrophilic bacilli were not significantly different, suggesting that the survival mechanism encompasses several ways via which acidophilic bacteria adapt to low pH (Richard and Foster, 2004).

A large number of DNA and protein repair genes in acidophiles contribute to preservation of pH homeostasis (Dopson et al., 2005). A DNA-binding protein in starved cells (Dps), which protects DNA from reactive oxygen species (ROS) generated by the Fenton reaction, plays a significant role when cells are exposed to oxidative and nutritional stress. Studies showed that the *E. coli* O157:H7 wild-type strain survives better than *dps*::*nptI* mutants in the initial 1 h of exposure to acidic conditions. Furthermore, complementation of wild type *dps* gene to *dps*::*nptI* mutants restored survival (Choi et al., 2000). However, available genome data showed that Dps is not present in *Acidiphilium*, suggesting that Dps synthesis does not occur by all alkane-degrading acidophiles for surviving under acidic conditions.

Upregulation of an outer membrane porin (Omp40) in *Acidithiobacillus ferrooxidans* was observed when pH was shifted from 3.5 to 1.5 (Amaro et al., 1991). Further studies illustrated that the positively charged L3 loop of the porin protein controls pore size and ion selectivity at pH 2.5 (Guiliani and Jerez, 2000). In addition, the decrease in membrane fluidity of *E. coli* O157:H7 may confer resistance to protons under acidic conditions (Yuk and Marshall, 2004). When lipids of acidophilic strains were adapted to acidic condition, palmitic acid (C_16:0_) concentrations increased, whereas that of *cis*-vaccenic acid (C_18_:1ω7c) decreased, resulting in reduced membrane fluidity, which regulated porin pore size. Low membrane fluidity confers resistance to toxicity from hydrocarbons and acidic condition, but compromises alkane uptake.

### Thermophiles

Most proteins with catalytic and regulatory functions in thermophiles are thermostable and are structurally more compact than their mesophilic counterparts. Hyperthermostable proteins in *Thermococcus onnurineus* NA1, hydperthermophilic archaea, incubated at 100°C include intracellular protease I, thioredoxin reductase, triosephosphate isomerase, putative hydroperoxide reductase, proteasome, and translation initiation factors (Yun et al., 2011). Furthermore, transcriptomic and proteomic analyses of thermophiles revealed that many thermostable proteins of certain categories [heat stable proteins, chaperonin, metalloenzyme, putative ribosomal associated proteins, and superoxide dismutase (SOD)] are translated at high temperatures, implying that they are essential for thermophile survival at those temperatures (Li et al., 2010; Shih and Pan, 2011; Wang W. et al., 2014). These proteins have large numbers of disulfide bonds and positively charged amino acids, such as lysine and arginine, which are critical for their stability (Brocchieri, 2004; Beeby et al., 2005; Ma et al., 2010). Additionally, *in silico* studies illustrated that disulfide bonds are ubiquitous among thermophiles. BLAST revealed the presence of nine genes encoding FAD-dependent pyridine nucleotide-disulfide oxidoreductase, a potential key enzyme in thermophilic intracellular disulfide-bond formation, in the thermophilic alkane degrader *Geobacillus* sp. Y4.1MC1 (Beeby et al., 2005). Increase in the number of small residues such as Gly, Ala, Ser, and Val, and decrease in the number of Cys and polar residues (Asp, Asn, Glu, Gln, and Arg) were observed in membrane proteins of many thermophiles, which increases stringent membrane hydrophobicity at high temperatures (Meruelo et al., 2012). The amino acid preference might occur in membrane proteins of thermophilic alkane degraders.

Proteome analyses in most thermophiles has demonstrated the presence of genes encoding highly thermostable proteins such as antioxidants, antitoxins, heat-shock proteins (Hsps), and enzymes involved in carbon metabolism pathways, including glycolysis (Wang Q. et al., 2015). Several antioxidants and antitoxins, such as the VapBC complex, are translated in response to high-temperature stress (Shih and Pan, 2011). Global transcriptomic analysis demonstrated that VapBC proteins, the virulence-associated proteins in the hyperthermophilic crenarchaeon *Sulfolobus solfataricus*, are induced at elevated temperatures ranging from 80–90°C. Furthermore, a Δ*vapBC S. solfataricus* knockout strain showed a highly altered transcriptomic profile and susceptibility to heat shock (Cooper et al., 2009). However, *S. solfataricus* VapBC homologues do not exist in any thermophilic alkane degrading bacteria (*Geobacillus* species, *Thermus brockianus*, and *Aeribacillus pallidus*) and archaea (*Archaeoglobus fulgidus* DSM 4304), indicating that the VapBC system is not a ubiquitous heat-shock defense mechanism. Small Hsps (sHSPs) bind to proteins and confer protection from denaturation under high temperatures (Li et al., 2012). Unlike Csp proteins, Hsp proteins are abundant in many thermophilic alkane degraders. Five Hsp-encoding genes are present in the *Geobacillus thermodenitrificans* NG 80-2 genome, and most *Geobacillus* species possess more than four genes. Thus, Hsp proteins in alkane-degrading thermophiles may play an important role in survival at high temperatures.

## Energy Production of Extremophiles During Alkane Metabolism

Most alkane-degrading extremophiles have ubiquitous aerobic or anaerobic alkane oxidation pathways represented by ω-oxidation or fumarate addition, respectively (**Figure 1**). It is assumed that most aerobic psychrophiles, halophiles, acidophiles, and thermophiles degrade alkanes through general alkane oxidation pathways, as demonstrated by the possession of AlkB, CYP, AlmA, and LadA-type alkane hydroxylases (Bertrand et al., 2013; Nie et al., 2013; Ivanova et al., 2014). However, some extremophiles oxidize alkanes via a unique pathway and produce energy via alternative pathways to avoid stress under harsh conditions.

### Novel Aerobic Alkane Degradation Systems in Extremophiles

The aerobic alkane degrader, *Dietzia* DQ12-45-1b, grown at 4–45°C, pH 6.0–12.0, and 0–20% (w/v) NaCl (Wang et al., 2011), possesses an *alkW1*-*alkX* system, which is co-expressed and induced by fatty acids (Liang et al., 2016). The proposed mechanism is that *alkX*, a TetR family regulator, competes with DNA polymerase at the *alkW1* promoter region and represses the expression of *alkW1*, an *alkB*-type alkane hydroxylase-rubredoxin fusion gene, in the absence of alkanes. However, fatty acids produced by alkane oxidation directly bind to AlkX, removing it from the *alkW1* promoter when alkanes are present, indicating that this system is energy-cost effective. Furthermore, phylogenetic and gene alignment analyses showed that *alkW1*-*alkX* may be ubiquitous in Actinobacteria such as *Rhodococcus* and *Mycobacterium*. Thus, *alkW*-*alkX* system confer benefits to Actinobacteria living in harsh conditions characterized by high pH, osmotic stress, and lack of nutrients (Liang et al., 2016).

Recent genome sequencing analysis revealed that the moderate halophile, *Amycolicicoccus subflavus* DQS3-9A1^T^ possesses four different alkane hydroxylation systems (propane monooxygenase, AlkB, CYP, and LadA) (Nie et al., 2013). A previously described halophile, *Alcanivorax dieselolei* B-5, harbors multiple copies of alkane hydroxylases (two AlkB-, one CYP153-, and AlmA-type alkane hydroxylase) and showed broad range of alkane-degrading capability (C_5_–C_36_) (Liu et al., 2011). Furthermore, the available complete genome sequence of *Marinobacter aquaeolei* VT8 shows the existence of three AlkB-, two CYP153-, and one AlmA-like alkane hydroxylase enzymes in the VT8 genome. Although the evolutionary reasons for possession of multiple alkane hydroxylase systems are not yet clear, it probably facilitates utilization of diverse hydrocarbon sources by halophilic bacteria under high saline environments. Illumination and addition of casamino acid as an organic fertilizer were tested to measure biodegradation efficiency of the extremely halophilic archaeal strains, *Haloferax*, *Halobacterium*, and *Halococcus* in highly saline soil (>22% salinity) and pond water (>16% salinity), which resulted in significantly enhanced biodegradation by halophilic archaea in the presence of casamino acid and illumination (Al-Mailem et al., 2012). It was hypothesized that archaea in hypersaline environment can synthesize ATP using a red pigment, perhaps a bacteriorhodopsin-like system, under low oxygen tension. Casamino acid also confers better growth and degradation of halophilic archaea, indicating promotion of biodegradation efficiency by addition of organic nitrogen source. Further studies are required to understand enhanced hydrocarbon degradation by haloarchaea in the presence of light and N source.

### Alternative Pathways Producing Energy in Extremophiles

The glyoxylate shunt in the mesophilic alkane-degrading bacteria, *Acinetobacter oleivorans* DR1, is activated to generate energy during triacontane metabolism (Park et al., 2017). The glyoxylate shunt requires isocitrate lyase (encoded by *aceA*) and malate synthase (encoded by *aceB* or *glcB*), which are activated under oxidative stress or in the presence of C_2_ carbon sources (Ahn et al., 2016). Among psychrophiles, upregulation of glyoxylate cycle-related genes in *Nesterenkonia* sp. AN1, isolated from Antarctic soil, was observed at 5°C. Furthermore, a large number of glyoxylate shunt-related genes belonging to the psychrophilic bacteria *Colwellia* and *Neptuniibacter* were observed in samples from the area surrounding the Deepwater Horizon oil spill (Rivers et al., 2013). In *Colwellia maris*, the expression level of *icl* was higher at 0°C than at 15°C (Watanabe et al., 2002), implying that *Colwellia* possess the advantage of surviving in cold environment by activating the glyoxylate shunt during alkane degradation.

Little is known about complete energy producing metabolism of alkane-degrading psychrophiles. The presence of methylglyoxal synthase in psychrophilic bacteria, *Exiguobacterium sibiricum*, was proposed as an important alternative catabolic pathway for glyceraldehyde phosphate (Rodrigues et al., 2008). In addition, the glyoxalase family proteins in *Planococcus halocryophilus* Or1 have been emphasized as key enzymes for utilization of carbon sources under cold environments. In addition, repression of energy metabolism at low temperature (-15°C) in *Planococcus halocryophilus* Or1, but increased expression of succinic semialdehyde dehydrogenase, alcohol dehydrogenase, and several oxidoreductases indicate maintenance of energy metabolism and ATP levels (Mykytczuk et al., 2013). Similarly, transcriptomic analysis of *Pseudomonas extremaustralis* showed activation of the ethanol oxidation pathway, involving a pyrroloquinoline quinone (PQQ)-dependent ethanol dehydrogenase, cytochrome c550, and an aldehyde dehydrogenase (encoded by *exaA*, *exaB*, and *exaC*, respectively) although genes associated with tricarboxylic acid (TCA) and cytochrome synthesis are repressed under cold conditions (Tribelli et al., 2018). Proteomics study suggested that enzymes participating in the fatty acid degradation pathway (β-oxidation) were more abundant at low temperatures (4–10°C) in cold adapting marine bacterium, *Sphingopyxis alaskensis* under artificial seawater medium (Ting et al., 2010). Cold adaptation strategy which uses fatty acid metabolism was also observed in *Pseudomonas putida* KT2440 at 10°C in Luria-Bertani (LB) medium. Analyses of proteomics and RNA sequencing data in KT2440 strain showed upregulation of the 2-methylcitrate and branched amino acid degradation pathways under cold temperature, implying conversion of propionate or propionyl-CoA to succinate and pyruvate via 2-methyl citrate under cold condition (Fonseca et al., 2011).

Proteomic analysis of the halophilic bacteria, *Alcanivorax borkumensis* SK2, also showed upregulation of AceA (ABO_2741) and GlcB (ABO_1267) and downregulation of TCA cycle enzymes, including isocitrate dehydrogenase (*icd*, ABO_1281) and 2-oxoglutarate dehydrogenase (*lpdG*, ABO_1494), when bacteria were grown in hexadecane-supplemented media, suggesting that the glyoxylate shunt is the main pathway for hexadecane metabolism (Sabirova et al., 2006). Similarly, another strategy for adapting to high temperatures involves a shift in the carbohydrate metabolism pathways. Transcriptomic and proteomic analysis of *Thermus filiformis* at 63°C, 70°C, or 77°C revealed that oxidative stress generated by high temperature induced genes related to the pentose phosphate (PP) pathway, whereas genes participating in glycolysis and the TCA cycle were downregulated (Mandelli et al., 2017), indicating evasion of ROS generation. In addition, metabolomic analysis revealed accumulation of ROS scavengers such as oxaloacetate and α-ketoglutarate, indicating preservation of ROS homeostasis. It has been reported that the PP pathway, glyoxylate cycle, and ROS defense systems are essential for detoxifying petroleum hydrocarbons (Feng et al., 2007; Kato T. et al., 2009; Shih and Pan, 2011). Thus, exposure to heat shock probably assists the growth of thermophilic alkane degraders at high temperatures in the presence of alkanes. The same pattern was observed in the genomic and proteomic analysis of thermophilic bacteria *Geobacillus thermodenitrificans* NG80-2, which showed activated glyoxylate shunt instead of the TCA cycle in the presence of hexadecane (Feng et al., 2007).

The interface between alkane degraders and hydrocarbon compounds may form microoxic or anoxic niches because of hydrophobicity (Suter et al., 2017). In addition, alkane oxidation generates excessive acetyl-CoA (Sabirova et al., 2006; Schönfeld and Wojtczak, 2016; Park et al., 2017), creating appropriate conditions for aerobic fermentation, which is also called the Crabtree effect (Zhou et al., 2017). Acetate assimilation via the Pta-AckA pathway occurs in the presence of long-chain alkanes (triacontane), producing ATP (Park et al., 2017). Although studies on the Pta-Ack pathway during alkane metabolism are limited, evidence shows that extremophiles can synthesize ATP via the Pta-AckA pathway. Phenotypic analysis of *Psychrobacter arcticus* 273-4 shows that acetic acid can be diffused into cells without energy consumption, whereas energy-consuming glucose could not be utilized at low temperatures (Bergholz et al., 2009). In addition, transcriptomic and proteomic analysis of *Psychrobacter* sp. PAMC 21119, which was grown under cold conditions, revealed upregulation of acetyl-CoA metabolism, although the central energy production and conversion pathways were downregulated (Koh et al., 2017). *Geobacillus thermodenitrificans* NG80-2 harbors *pta* and *ackA*, which are indicative of possible methods of ATP synthesis during alkane assimilation (Feng et al., 2007). Methanogenic archaea and bacteria also possess *pta* and *ackA* genes. Furthermore, methanogenic and halophilic archaea have the same ancestral Pta lineage (Barnhart et al., 2015), implying the possibility of acetate production via the Pta-AckA pathway during alkane metabolism at high salinity.

### Novel Alkane Degradation Pathways Under Anaerobic Conditions

Although many alkane hydroxylase genes involved in aerobic alkane degradation were detected in extremophiles, anaerobic alkane metabolism may have advantages, particularly because extreme environments confer oxidative stress on bacteria. The thermophilic sulfate-reducing archaeon, *Archaeoglobus fulgidus* strain VC-16, which degrades C_10_–C_21_ alkanes with thiosulfate or sulfate as a terminal electron acceptor, possess pyruvate formate lyase (encoded by *pflD*), which is homologous to alkylsuccinate synthase (encoded by *assA*). Phylogenetic analysis showed that homologs of PflD and PflC mainly belong to bacteria such as Firmicutes, δ-proteobacteria, γ-proteobacteria, and Actinobacteria, indicating that these genes are acquired from bacteria via HGT. Furthermore, three-dimensional simulation suggested that PflD might be a potential alkylsuccinate synthase, which is supported by the upregulation of *pflD* gene expression in the presence of hexadecane but not fatty acid (Khelifi et al., 2014). An anaerobic hyperthermophile, *Geoglobus acetivorans*, isolated from deep-sea hydrothermal vents, requires ferric iron (Fe (III)) as an electron acceptor during alkane degradation. Genome analysis revealed that this archaeon also harbors close homologs of *Archaeoglobus fulgidus* PflC and PflD (69 and 57% identity, respectively). However, Gace_0240 (homolog to PflC in *Archaeoglobus fulgidus*) shares features with alkyl and benzylsuccinate synthases, rather than with pyruvate formate lyase-activating enzymes (Mardanov et al., 2014).

A methanogenic culture, enriched from freshwater hydrocarbon-contaminated aquifer sediments, consumed octacosane (C_28_) and generated methane. *Smithella* was predominant and *assA* was abundant in the presence of octacosane, furthermore, phylogenetic analysis showed that *assA* copies in the culture were closely related to *assA* of *Smithella*, indicating that fumarate addition in octacosane is mainly performed by *Smithella.* Interestingly, the detection of α,ω-dicarboxylic acids in the octacosane-only media implies simultaneous activation by fumarate addition, although the mechanism remains unclear (Oberding and Gieg, 2017). Another alkane degradation pathway has been suggested in *Verrucomicrobia* methylotrophs, which grow optimally at pH 2.0–2.5 and consume methane. Interestingly, analysis of the draft *Verrucomicrobia* genome detected genes encoding pMMO, which is homologous to the pMMO in methanotrophic proteobacteria. However, genes for subsequent methanol and formaldehyde oxidation were incomplete or missing, suggesting that the bacterium uses novel methylotrophic pathways (Dunfield et al., 2007). Further analysis showed that three *pmoA* genes in *Verrucomicrobia* were located in a different clade of proteobacterial *pmo* homologs, indicating divergent evolution with proteobacterial methanotrophs. Recent metatranscriptome, metaproteome, and metagenome analyses of *Candidatus Syntrophoarchaeum butanivorans* showed that genes encoding β-oxidation enzymes, carbon monoxide dehydrogenase, and reversible C_1_ methanogenesis enzymes [reverse methyl coenzyme M (Mcr)] are also expressed, indicating that enzymes for complete butane metabolism exist in *Ca. Syntrophoarchaeum butanivorans.* Furthermore, ultra-high resolution mass spectrometry data showed that *Ca. Syntrophoarchaeum butanivorans* generates alkyl-coenzyme M. Phylogenetic analysis showed that MCR was present in the draft genomes of *Bathyarchaeota* and pan-genome of uncultivated *Hadesarchaea*, indicating that uncultivated alkane degraders may perform non-methane alkane degradation (Laso-Pérez et al., 2016).

## Biotechnological Application of Extremophiles in Bioremediation

*In situ* bioremediation of petroleum, mainly applied to soil and groundwater, includes biostimulation and bioventing. Biostimulation was recently performed in desert soil near the Arabian Gulf region (Al-Mailem et al., 2015). Community analysis revealed that 19 thermophilic hydrocarbonoclastic bacterial taxa were present in the soil sample. Furthermore, predominant species belonged to *Amycolatopsis*, *Chelativorans*, *Isoptericola*, *Nocardia*, *Aeribacillus*, *Aneurinibacillus*, *Brevibacillus*, *Geobacillus*, *Kocuria*, *Marinobacter*, and *Paenibacillus*, which showed better growth and hydrocarbon consumption rate at 50°C than at 30°C. However, enhanced growth and hydrocarbon consumption were observed after supplementation with Ca^2+^ (∼2.5 M CaSO_4_) or addition of 8% w/v dipicolinic acid (DPA), implying that Ca^2+^ and DPA-mediated increased heat resistance of alkane degraders may stimulate biodegradation at high temperatures (Radwan et al., 2017). A bioelectrochemical system was recently applied to marine sediments for hydrocarbon biodegradation, wherein a graphite rod (snorkel) is placed between the anoxic contaminated sediment and the O_2_-containing water. The snorkel acts as an electron acceptor and shuttles electrons generated by anaerobic hydrocarbon oxidation using reducing elements, such as S^2^ and Fe^2+^. Electrons move to the water phase along the graphite and fuse with oxygen and protons to form water (Viggi et al., 2015). This device serves as a sustainable respiratory electron acceptor in anaerobic oxidation of hydrocarbons, and can stimulate hydrocarbon degradation by SRB by scavenging toxic sulfide (Mapelli et al., 2017). Psychrophilic bacteria can potentially be used to degrade organic pollutants from soil and water at low temperatures, mainly, psychrophilic petroleum degraders such as *Acinetobacter, Arthrobacter, Colwellia, Cytophaga, Halomonas, Marinobacter, Marinomonas, Pseudoalteromonas, Oleispira, Rhodococcus*, and *Shewanella* (Brakstad et al., 2008). *Acinetobacter* spp. isolated from oil-polluted soils of King George Island were inoculated into a microcosm of contaminated soil, resulting in reduction of contaminant concentration by 75% after 50 days, compared to NP addition, which had no significant influence (Ruberto et al., 2003). Rhoder, composed of hydrocarbon-degrading bacteria *Rhodococcus ruber* and *R. erythrococcus*, oil, and gas, was used for removing oil pollution (Murygina et al., 2000). Over 99% bioremediation rate was obtained in open aquatic surfaces with light contamination (<20 g L^-1^); however, primary mechanical collection of oil and triple Rhoder treatment resulted in a 94% bioremediation rate in heavily polluted aquatic systems (thickness of oil film > 3 mm) and wetland (2,000 m^2^) in Urai, indicating that complex bioremediation strategies for effective oil-degradation are required.

Biopile bioremediation involves excavation of polluted soil, followed by supply of nutrients and air for increasing microbial hydrocarbon degradation and promoting bioremediation. Biopile remediation has recently been applied to polluted extreme environments such as cold regions (Gomez and Sartaj, 2014; Dias et al., 2015; Whelan et al., 2015; Martínez Álvarez et al., 2017). In a recent study, a previously suggested nutrient ratio (C:N:P ratio of 100:17.6:1.73) for biological activity (as FDA levels) was used to treat 0.4-ton geomembrane-covered biopiles from diesel fuel storage tanks of Antarctic expedition (Martínez Álvarez et al., 2017). Results showed a higher hydrocarbon removal rate (75.9%) in biostimulated soil within 40 days than in the control biopile (49.5%). In another study, an optimized biopile condition was obtained using surface response methodology (RSM) - a combination of 3 mL m^-3^ microbial consortia and 5% mature compost. After 94 days, 90.7% petroleum hydrocarbon reduction was observed, whereas hydrocarbon was reduced by only 56% in control soil (excavated from Val-des-Bois, in the Outaouais region of Quebec, Canada) between 1 and 8°C, indicating synergistic interaction between bioaugmentation and biostimulation for bioremediation enhancement. Additionally, biostimulation of Antarctic soils with fishmeal reduced 71% of total hydrocarbon and facilitated a bacterial community shift, increasing Actinobacteria over the 50-day study period (Dias et al., 2015). Landfarming has also been demonstrated as an effective bioremediation method in polar regions, which involves aggressive soil tilling and selective fertilization with a mixture of fertilizers (ammonium phosphate and urea) over a span of 70 days (McCarthy et al., 2004). However, soil concentrations of diesel range organics in northeast (380 mg kg^-1^) and southwest soil (430 mg kg^-1^) were already reduced to the target concentrations (500 mg kg^-1^) at 31 and 55 days, respectively.

## Concluding Remarks

Although many alkane degraders living in extreme environment has been isolated and characterized, there is a great number of uncharacterized extremophiles that appear to be alkane-degraders, such as *Brachybacterium*, *Idiomarina*, and *Leifsonia*. Thus, further investigation of such novel alkane-degrading extremophiles will provide new metabolic pathways and survival strategies under harsh environments. Recent studies on discovery of new alkane metabolic systems in aerobic-, anaerobic extremophiles, such as AlkW1-AlkX system, Pfl homologues, and alkyl-coenzyme M indicate that there are several questions to be solved regarding alkane degradation metabolism. The evidences demonstrated the activation of several alternative pathways, such as glyoxylate shunt, Pta-AckA, or alcohol/fatty acid metabolism, instead of TCA cycle when extremophiles survive under extreme condition, implying that they are beneficial during alkane degradation. Because of high adaptability, strong stress resistance, and unique biodegradation capability, extremophiles are promising bioremediation-reagents to clean up polluted environments with low cost and high efficiency.

## Author Contributions

CP and WP designed and coordinated the study. CP collected the data. CP wrote the first complete draft of the manuscript. WP provided substantial modifications. All authors contributed to and approved the final version of the manuscript.

## Conflict of Interest Statement

The authors declare that the research was conducted in the absence of any commercial or financial relationships that could be construed as a potential conflict of interest.

## Footnotes

1 https://www.ncbi.nlm.nih.gov/

2 https://img.jgi.doe.gov/
