# Supplementary material for: Survival and Energy Producing Strategies of Alkane Degraders Under Extreme Conditions and Their Biotechnological Potential
Source: Front Microbiol. 2018 May 25;9:1081. doi: 10.3389/fmicb.2018.01081 (PMC5992423; doi:10.3389/fmicb.2018.01081)
Supplement: Supplementary file 1 [file Table_1.DOCX]

**Table S1** Information of putative alkane degraders under extreme condition introduced in this study.

| Extremophiles | Isolation site | Alkane oxidizer | Genome | | Genome Size (bp) | Research Keyword | Reference |  |
| --- | --- | --- | --- | --- | --- | --- | --- | --- |
| Halophile | | | | | | | |  |
| *Brachybacterium* sp. P6-10-X1 | Deep-sea sediments | CYP, LadA | Y | 4,385,603 | | Genome analysis | Zhao et al (2017) |  |
| *Kordiimonas gwangyangensis* | Gwangyang bay sediment | AlkB, CYP | Y | 4,082,245 | | Phenotype characterization | Kwon et al (2005) |  |
| *Alcanivorax* sp. DG881 | Shellfish | AlkB, CYP, AlmA | Y | 3,804,728 | | Ecological interaction | Matsumoto et al (2017) |  |
| *Alcanivorax pacificus* W11-5 | Marine sediment | AlkB, CYP, AlmA | Y | 4,137,438 | | Genome analysis | Lai and Shao (2012) |  |
| *Alcanivorax dieselolei* B5 | Sea water and deep-sea sediment | AlkB, CYP, AlmA | Y | 4,928,223 | | Genome analysis, hydrocarbon degradation | Liu and Shao (2005); Lai et al (2012); Liu et al (2011); Wang and Shao (2014) |  |
| *Alcanivorax borkumensis* SK2 | Sea water/sediment | AlmA | Y | 3,120,143 | | Genome analysis, hydrocarbon degradation, triacylglycerol biosynthesis, water pressure adaptation | Schneiker et al (2006); Kalscheuer et al (2006); Sabirova (2016); Scoma et al (2016); |  |
| *Amycolicicoccus subflavus*  DQS3-9A1 | Saline soil | AlkB, CYP, AlmA | Y | 4,738,809 | | Genome analysis, Phenotype characterization | Wang et al (2010); Cai et al (2011) |  |
| *Marinobacter aquaeolei* VT8 | Offshore oil platform | AlkB, CYP, AlmA | Y | 4,779,762 | | Protein characterization, biosynthesis | Willis et al (2011); Lenneman (2013) |  |
| *Marinobacter daepoensis*  DSM 16072 | Intertidal zone | AlkB, AlmA | Y | 3,835,704 | | Phenotype characterization | Yoon et al (2004) |  |
| *Marinobacterium lutimaris*  DSM 22012 | Intertidal zone | CYP | Y | 5,568,333 | | Phenotype characterization | Kim et al (2010) |  |
| *Marinobacter hydrocarbonoclasticus* SP17 | Sea water | AlkB, CYP, AlmA | Y | 3,989,480 | | Characterization of nitric/nitrous oxide reductase, hydrocarbon degradation, biofilm | Dell'Acqua et al (2012); Mounier et al (2014); Branchu et al (2017) |  |
| *Salinisphaera shabanensis* E1L3A | Brine-seawater | AlkB, CYP | Y | 3,760,335 | | Genome analysis | Antunes et al (2003) |  |
| *Marinobacter algicola* DG893 | Sea water | AlkB, AlmA | Y | 4,413,003 | | Iron uptake and regulation, protein characterization | Romano et al (2013); Barker et al (2015) |  |
| *Paracoccus saliphilus*  DSM 18447 | Saline-alkali soil | AlkB, CYP | Y | 4,570,660 | |  |  |  |
| Psychrophile | | | | | | | |  |
| *Glaciecola punicea*  DSM 14233 | Sea ice diatom assemblages | AlkB, AlmA | Y | 3,076,861 | | Solutes tolerance | Chin et al (2010) |  |
| *Glaciecola nitratireducens* FR1064 | Seawater | AlkB, AlmA | Y | 4,134,229 | | Genome analysis, phenotype characterization | Bian et al (2011); Baik et al (2006) |  |
| *Octadecabacter arcticus* 238 | Freshwater | AlkB, CYP | Y | 5,478,249 | | Genom plasticity, phenotype characterization | Vollmers et al (2013); Gosink et al (1997) |  |
| *Octadecabacter antarcticus* 307 | Freshwater | AlkB, CYP | Y | 4,875,481 | | Stress regulation, phenotype characterization | Cude et al (2015); Gosink et al (1997) |  |
| *Sulfitobacter mediterraneus* DSM 12244 | sea water | AlkB, CYP | Y | 4,099,776 | | Phenotype characterization | Pukall et al (1999) |  |
| *Algoriphagus antarcticus*  DSM 15986 | Antarctica lake | AlkB | Y | 5,092,408 | | Phenotype characterization | Rau et al (2012) |  |
| *Paraglaciecola psychrophila* 170 | Sea ice | CYP, AlmA | Y | 5,413,691 | | Genome analysis | Yin et al (2013) |  |
| *Pseudomonas fragi* A22 |  | CYP, LadA | Y | 5,061,208 | | Genome analysis | Mei et al (2011) |  |
| *Pseudomonas fragi* B25 |  | LadA | Y | 5,000,569 | |  |  |  |
| *Pseudomonas frederiksbergensis* | Coal gasification site | CYP, LadA | Y | 6,394,336 | | Genome analysis | Andersen et al (2000) |  |
| *Psychrobacter cryohalolentis* K5 | Fresh water | AlmA | Y | 3,101,097 | | Protein characterization, polysaccharide, proteomic analysis | Novototskaya-Vlasova et al (2013); Novototskaya-Vlasova et al (2015); Petrovskaya et al (2015); Kondakova et al (2012);  Bakermans et al (2007) |  |
| *Rhodococcus sp.* AW25M09 | Atlantic Hagfish | AlkB, CYP, AlmA, LadA | Y | 5,642,031 | | Protein characterization | De Santi (2014) |  |
| *Rhodococcus* sp. JG-3 | Atlantic Hagfish | AlkB, CYP, AlmA, LadA | Y | 5,286,918 | | Comparative genome analysis | Goordial et al (2016) |  |
| *Terriglobus saanensis* SP1PR4 | Tundra soil | CYP | Y | 5,095,226 | | Genome analysis, phenotype characterization | Mannisto (2011); Rawat (2012) |  |
| Acidophile | | | | | | | |  |
| *Acidiphilium cryptum* JF-5 | Coal mine lake sediment | AlkB | Y | 3,963,080 | | Compatible solutes, metal reduction, PHB synthesis | Moritz et al(2015); Magnuson et al (2010); Küsel et al (2002); Cummings (2007); Küsel et al (1999); Xu et al (2010) |  |
| *Acidiphilium multivorum* AIU301 | Pyritic acid mine drainage | AlkB | Y | 4,214,744 | | Genome analysis | Ullrich et al (2015) |  |
| *Acidiphilium* sp. PM | Heavy metal-rich waters | AlkB | Y | 3,929,465 | | Genome analysis | San Martin-Uriz et al(2011) |  |
| *Acidiphilium* sp. JA12-A1 | Acid mine drainage | AlkB | Y | 4,184,331 | | Genome analysis | Ullrich et al (2015) |  |
| Thermophile | | | | | | | |  |
| *Geobacillus* sp. Y4.1MC1 | Hot Spring | LadA | Y | 3,911,947 | | Genome analysis | Brumm et al (2015) |  |
| *Geobacillus thermodenitrificans* NG80-2 | Oilfield | LadA | Y | 3,608,012 | | Genome and proteome analysis, hydrocarbon degradation, hydrolysis of xylan, enzyme turnover, ornithin production, superoxide dismuatases thermostability | Huang et al (2017); Lintuluoto (2016); Huang et al (2016); Wang (2014); Li et al (2012); Feng et al (2007) |  |
| *Parageobacillus thermoglucosidasius* C56-YS93 | Hot Spring | LadA | Y | 3,993,793 | | Genome analysis, Ethanol production | Brumm et al (2015); Zhou (2016) |  |
| *Geobacillus thermoleovorans* CCB_US3_UF5 | Thermal springs | LadA | Y | 3,596,620 | | Genome analysis | Muhd Sakaff et al (2012) |  |
| *Geobacillus sp.* GHH01 | Soil | LadA | Y | 3,583,134 | | Genome analysis | Wiegand et al (2013) |  |
| *Aeribacillus pallidus* 8m3 | High-temperature oilfields | LadA | Y | 3,815,829 | | Genome analysis | Poltaraus (2016) |  |
| *Thermus brockianus* | Hot springs | CYP | Y | 2,483,116 | | Phenotype characterization | Williams et al (1995) |  |
| *Aeribacillus pallidus* GS3372 | Deep geothermal reservoir | CYP, LadA | Y | 4,985,863 | | Genome analysis | Filippidou (2015) |  |
| Anaerobe | | | | | | | |  |
| *Methanoculleus marisnigri* JR1 | Marine sediment | Mcr | Y | 2,478,101 | | Genome analysis | Anderson et al (2009) |  |
| *Methanoculleus* sp. MH98A | Methane hydrate sediment | Mcr | Y | 2,542,436 | | Genome analysis | Dabir et al(2014) |  |
| *Methanoculleus bourgensis* MS2 | Sewage sludge digester | Mcr | Y | 2,789,773 | | Genome analysis | Maus et al (2015) |  |
| *Methanocorpusculum bavaricum* DSM 4179 | Wastewater pond | Mcr | Y | 1,706,782 | | Phenotype characterization | Zellner et al (1989) |  |
| *Methanoculleus sp.* MAB1 | Methane forming sludges | Mcr | Y | 2,859,299 | | Syntrophic acetate oxidation, metagenomics | Schnürer et al (1999); Kougias et al (2017) |  |
| *Methanosphaerula palustris* E1-9c | Rich minerotrophic fen | Mcr | Y | 2,922,917 | | Genome analysis | Cadillo-Quiroz (2015) |  |
| *Methanolacinia petrolearia* DSM 11571 | Offshore oil field | Mcr | Y | 2,843,290 | | Genome analysis | Brambilla et al (2010) |  |
| *Methanoregula formicica* SMSP | Methanogenic sludge | Mcr | Y | 2,820,858 | | Phenotype characterization | Yashiro et al (2011) |  |
| *Methanoregula boonei* 6A8 | Acidic peat bog | Mcr | Y | 2,542,943 | | Phenotype characterization | Brauer et al (2015) |  |
| *Methanolinea tarda* NOBI-1 | Anaerobic digested sludge | Mcr | Y | 2,052,856 | | Genome analysis | Yamamoto et al (2014) |  |
| *Methanofollis liminatans* GKZPZ | Waste water | Mcr | Y | 2,475,100 | | Phenotype characterization, secondary alcohol metabolism | Zellner et al (1999); Zellner et al (1989) |  |
| *Methanomicrobium mobile* DSM 1539 | Bovine rumen | Mcr | Y | 1,711,791 | | Light sensitivity | Olson et al (1991) |  |
| *Methanospirillum hungatei* JF-1 | Sewage sludge | Mcr | Y | 3,544,738 | | Mercury methylation; coculture | Yu et al (2013); de Bok et al (2005) |  |
| *Methanoplanus limicola* M3 | Drilling swamp mud | Mcr | Y | 3,200,946 | | Genome analysis | Göker et al (2014) |  |
| *Methanolobus psychrophilus* R15 | Wetland soil | Mcr | Y | 3,072,769 | | Proteomics, genomics, transcriptomics | Chen (2015); Chen (2012) |  |
| *Methanolobus tindarius* DSM 2278 | Sea water sediment | Mcr | Y | 3,151,883 | | Quantitation of coenzyme M | Elias et al (1999) |  |
| *Methanosaeta harundinacea* 6Ac | Anaerobic sludge | Mcr | Y | 2,571,034 | | Transcriptomics, phenotype characterization | Ma et al (2006); Zhou et al (2015); Li et al (2015) |  |
| *Methanosaeta thermophila* PT | Anaerobic sludge | Mcr | Y | 1,879,471 | | Filaments, transcriptomics | Dueholm et al (2015); Kato et al (2014) |  |
| *Methanosaeta concilii* GP-6 | Anaerobic sludge | Mcr | Y | 3,026,645 | | Immobilization patterns | Schmidt (1999) |  |
| *Syntrophus gentianae* DSM 8423 | Anoxic sewage digestor | AssA | Y | 3,716,692 | | PAH metabolism, Genome analaysis | Schocke et al (1998); Wallrabenstein et al (1995) |  |
| *Smithella sp.* ME-1 |  | AssA | Y | 2,907,642 | | Alkane degradation | Tan et al (2014) |  |
| *Smithella sp.* F21 | Oil sands tailings pond |  | Y | 1,633,602 | | Comparative genome analysis | Tan et al (2014) |  |
| *Smithella sp.* SCADC | Oil field water | AssA | Y | 3,198,701 | | Metagenomic analysis | Tan et al (2013) |  |
| *Desulfosarcina variabilis Montpellier* | Marine black mud | AssA | Y | 9,415,595 | |  |  |  |
| *Desulfosarcina* sp. BuS5 | Gas cold seeps | AssA | Y | 3,600,571 | | Alkane degradation | Jaekel et al (2013) |  |
| *Desulfococcus oleovorans* Hxd3 | Oilfield mud | AssA | Y | 3,944,167 | | Alkane degradation | So et al (2003) |  |
| *Archaeoglobus fulgidus VC-16* | Heated sea floor | AssA | Y | 2,178,400 | | Genome analysis, carbon monoxide metabolism, alkane degradation, heat-shock response | Klenk et al (1997); Hocking (2015); Khelifi et al (2014); Rohlin et al (2005) |  |
